# Antimicrobial Resistance as a Global Public Health Challenge: Epidemiological Burden, Bioethical Dimensions and Emerging Therapeutic Strategies

**DOI:** 10.3390/idr18040070

**Published:** 2026-07-09

**Authors:** Christos Ntais, Ioanna P. Chatziprodromidou

**Affiliations:** 1 Epidemiology Program, School of Science and Technology, Hellenic Open University, 26335 Patras, Greece; 2 Department of Public Health, Medical School, University of Patras, 26504 Patras, Greece

**Keywords:** antimicrobial resistance, public health, epidemiology, bioethics, infectious diseases, antibiotics

## Abstract

Background/Objectives: Antimicrobial resistance (AMR) is a major global public health threat, compromising prevention and treatment of infectious diseases. This narrative review examines AMR as a multifactorial and transnational crisis through epidemiological, One Health, social and bioethical perspectives, and discusses emerging non-antibiotic preventive and therapeutic strategies. Methods: PubMed and Scopus were searched using terms related to AMR, epidemiology, public health, surveillance, One Health, bioethics, equity and alternative therapies. Peer-reviewed medical and public health articles were considered, together with selected reports from international organizations and public health agencies. Results: AMR is driven by inappropriate antibiotic use in human medicine, livestock, aquaculture and agriculture, combined with weaknesses in infection prevention, stewardship, environmental control and surveillance. Epidemiological evidence shows a substantial global burden, marked regional inequalities in resistance patterns, surveillance capacity and policy response, and major consequences, including increased mortality, prolonged hospitalization, rising healthcare costs and disproportionate effects on vulnerable populations. Key bioethical concerns include collective responsibility, equitable access to effective treatment, stewardship, global justice and intergenerational accountability. Emerging non-antibiotic strategies vary in translational maturity: vaccines and selected microbiome-based interventions have preventive or supportive roles in defined settings, bacteriophage therapy is used mainly in compassionate or specialized contexts, and many antimicrobial peptides and nanotechnology-based platforms remain experimental or early translational. Conclusions: AMR requires coordinated global action grounded in One Health, strong public health systems, integrated surveillance, responsible antimicrobial use and sustained innovation. Effective containment must also address social inequalities, ethical stewardship, equitable access to diagnostics and treatment, and responsibility toward future generations.

## 1. Introduction

Antimicrobial resistance (AMR) is one of the major threats to public health around the world [1,2,3]. As stated by the World Health Organization (WHO), the rising ineffectiveness of antimicrobials makes it difficult to treat everyday infections and will likely bring humanity back to a time where minor infections can potentially be lethal [1,2,3].

Resistance develops when bacteria either develop genetic mutations or obtain genes that allow them to resist antimicrobial agents that had previously worked [4,5]. Overuse and overprescription of antibiotics in people and livestock as well as inadequate healthcare systems along with travel and mobility across borders contribute to the process of developing resistance [1,2,3].

The crisis caused by AMR has many dimensions and extends beyond health alone. It is also an economic and social issue and, importantly, a bioethical concern. The ethical relevance of AMR arises because antibiotic use by one person, institution, or sector affects the treatment options available to others, including future generations. Thus, AMR raises questions of justice, solidarity, collective responsibility and equitable access to effective antimicrobials, diagnostics and infection-prevention measures. In 2019, an estimated 1.27 million deaths were directly attributable to bacterial AMR and 4.95 million deaths were associated with bacterial AMR [6]. More recent global forecasts suggest that annual AMR-attributable deaths could rise substantially by 2050, with an even larger number of deaths associated with resistant bacterial infections if coordinated interventions are not strengthened [7,8].

This paper examines the global nature of the current AMR crisis, analyzes some epidemiologic data from several countries and explores the bioethical issues surrounding the AMR crisis. Additionally, by presenting recent data and examples of how different countries have attempted to address the issue of AMR, it seeks to highlight both the challenges and the potential solutions for mitigating this crisis.

## 2. Materials and Methods

This paper represents a narrative review of the existing scientific literature on AMR as a global public health challenge, with emphasis on its epidemiological burden, major drivers, health and socioeconomic consequences, bioethical dimensions, One Health determinants and emerging non-antibiotic therapeutic approaches. The literature search was conducted using the PubMed and Scopus databases through April 2026. The search strategy combined keywords and Boolean operators, including (“antimicrobial resistance” OR “antibiotic resistance” OR “drug-resistant infection*” OR “multidrug-resistant bacteri*”) AND (“public health” OR “epidemiology” OR “surveillance” OR “disease burden” OR “morbidity” OR “mortality”) AND (“risk factor*” OR “antimicrobial stewardship” OR “infection control” OR “One Health” OR “wastewater” OR “aquaculture” OR “wildlife” OR “environment*”) AND (“bioethics” OR “equity” OR “collective responsibility” OR “intergenerational responsibility” OR “justice”) AND (“alternative therap*” OR “bacteriophage*” OR “antimicrobial peptide*” OR “immunotherap*” OR “vaccine*” OR “probiotic*” OR “microbiome” OR “nanotechnology” OR “rapid diagnostic*” OR “genomic surveillance” OR “artificial intelligence”).

Articles published in peer-reviewed international medical and public health journals were considered together with selected documents from international organizations and public health agencies, including the World Health Organization (WHO), European Centre for Disease Prevention and Control (ECDC), Centers for Disease Control and Prevention (CDC), Food and Agriculture Organization (FAO), United Nations Environment Programme (UNEP), World Organisation for Animal Health (WOAH) and World Bank. Inclusion criteria were (i) publication in English; (ii) primary emphasis on literature published between 2020 and 2026, with selected earlier landmark studies retained when necessary for conceptual, methodological, or epidemiological context; (iii) relevance to AMR in human health or to One Health pathways directly linked to human disease burden and control; and (iv) direct relevance to the thematic axes of the review. Grey literature, theses and dissertations, non-peer-reviewed conference proceedings and internal industry analyses were excluded, except for authoritative policy reports from international public health agencies.

Titles and abstracts were screened for thematic relevance and full texts were reviewed when they directly informed one or more sections of the paper. Because the manuscript is a narrative review rather than a systematic review or meta-analysis, no formal risk-of-bias assessment or pooled quantitative synthesis was performed. Evidence was prioritized according to relevance to the review question, recency, methodological robustness, geographic representativeness and the authority of the source.

## 3. Causes and Risk Factors for Antimicrobial Resistance

AMR is the result of an array of biological and non-biological elements. While it is a biological process, its development and spread have been further accelerated and increased by human actions, policy decisions and deficiencies within healthcare systems.

### 3.1. Overconsumption and Inappropriate Use of Antibiotics in Medicine

The single largest contributor to AMR is the misuse of antibiotics on a large scale, often without necessity or without appropriate medical supervision [9]. Estimates indicate that approximately 50% of antibiotics used globally were prescribed improperly or ineffectively according to WHO data [10]. Many countries still suffer from high rates of self-medicating, further increasing the selection pressures on emerging resistant bacterial strains [11].

### 3.2. Antibiotic Use in Agriculture and Livestock Farming

The routine use of antimicrobials to prevent disease and promote animal growth creates large numbers of resistant bacteria; these can then be transferred to people via the food supply, the environment or direct contact with infected animals [12,13]. For example, studies suggest that greater than half of all antibiotics used throughout the world are consumed by animals rather than humans [14].

### 3.3. Poor Adherence to Treatment

When patients discontinue their medication too early upon noticing an improvement in clinical symptoms or when they do not receive adequate doses, this creates an opportunity for partially resistant bacterial populations to develop into completely resistant populations [15,16].

### 3.4. Inadequate AMR Surveillance

It is estimated that only 45% of European Union (EU) member states conduct some form of AMR surveillance in every hospital in their country [17]. The inability to establish effective mechanisms to monitor AMR within both national systems and healthcare institutions limits the ability to implement targeted interventions.

### 3.5. Insufficient Infection Control

Although many health care systems implement standard infection prevention measures such as hand hygiene, instrument sterilization and patient isolation to reduce the risk of infection transmission between patients, failures in the consistent enforcement of these practices facilitate the transmission of resistant pathogens among hospitalized patients [18].

### 3.6. Delay in the Development of New Antibiotics

Investment in the development of new antibiotics has declined, largely due to economic disincentives faced by pharmaceutical companies [19]. As a result, clinicians are increasingly faced with managing multidrug-resistant infections despite a shrinking pool of effective treatment options. Consequently, the number of novel antibiotics progressing to late-stage clinical trials has significantly decreased [20].

### 3.7. Global Mobility and Cross-Border Spread

Globalization, international travel and world trade and commerce enable the rapid transnational spread of resistant bacterial strains, allowing them to move between regions with differing levels of healthcare preparedness and policy effectiveness [21,22].

### 3.8. Inequalities and Socioeconomic Factors

Disparities in access to healthcare services, clean water, sanitation infrastructure and medications significantly exacerbate the impact of AMR in low- and middle-income countries [23,24].

### 3.9. One Health, Environmental Reservoirs and Genetic Dissemination

**Figure 1:**
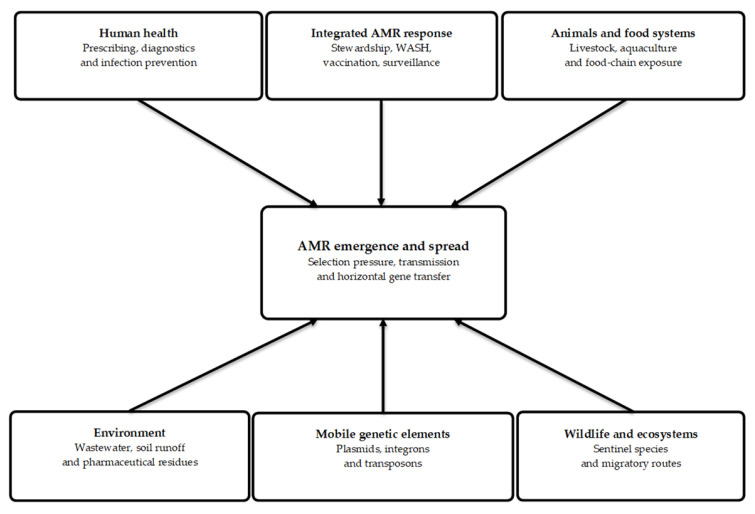
One Health pathways in antimicrobial resistance.

A strengthened One Health perspective is essential because AMR emerges and spreads through interconnected human, animal, food-system and environmental pathways [25,26,27,28] (Figure 1). Wastewater from hospitals, communities, farms and pharmaceutical production can concentrate antibiotic residues, resistant organisms and resistance genes, creating environmental reservoirs that facilitate dissemination through surface water, soil, irrigation and food chains [26,27]. Aquaculture and livestock systems may further amplify selective pressure when antimicrobials are used for treatment, prophylaxis, or growth promotion, while wildlife can act as sentinels and potential vectors of resistant bacteria across ecosystems and borders [25,26,27].

At the molecular level, mobile genetic elements, including plasmids, integrons and transposons, enable horizontal transfer of resistance determinants between bacterial species and ecological niches [28]. Therefore, AMR control cannot rely only on hospital-based stewardship. It also requires integrated surveillance across human, animal, food and environmental sectors; improved wastewater management; vaccination and biosecurity in animal production; reductions in unnecessary antimicrobial use; and equitable access to diagnostics, clean water, sanitation and effective medicines [25,26,27,28].

## 4. Epidemiological Data on Antimicrobial Resistance

With a growing number of patients suffering from infections caused by antimicrobial-resistant microorganisms, AMR represents one of the biggest threats to human health today. Available epidemiological data reveal substantial differences in both the magnitude of the problem and the ability to monitor and counter the threat globally [29]. A global burden analysis estimated 1.27 million deaths directly attributable to bacterial AMR and 4.95 million deaths associated with bacterial AMR [6]. More recent forecasts based on 1990–2021 trends suggest that annual attributable mortality could approach 1.91 million deaths by 2050, while associated deaths may be substantially higher, emphasizing the need for immediate prevention, diagnostics and access interventions [8]. WHO–Global Antimicrobial Resistance and Use Surveillance System (GLASS) data also show that resistance is reported across all WHO regions, although surveillance completeness and laboratory capacity remain uneven [30].

In the United States, AMR continues to rank as one of the leading causes of failed treatment using antimicrobials. Every year, an estimated 2.8 million infections caused by resistant pathogens occur, resulting in at least 35,000 deaths [31]. Furthermore, despite the advances in AMR prevention, resistance continues to increase in key pathogens such as *Candida auris* and carbapenem-resistant *Enterobacteriaceae*, reflecting the evolving and hard-to-control nature of the issue [31]. There is a large-scale surveillance system in place in the U.S., along with considerable government investment into the development of new antimicrobial therapies and prevention measures via national programs, including the “Antibiotic Stewardship Program” [32].

In the EU, a 2023 report by the ECDC reported approximately 33,000 deaths attributable to AMR in the previous two years, with nearly all cases resulting from hospital-acquired infections [33]. The annual economic impact of AMR is estimated at over €1.5 billion [33]. Resistance patterns vary considerably throughout the EU. For instance, countries located in southern and eastern Europe (such as Greece, Italy and Romania) demonstrate significantly higher resistance rates when compared with other EU nations, specifically for Gram-negative bacteria like *Klebsiella pneumoniae* and *Acinetobacter baumannii* [33].

Greece represents a particularly severe case within the EU context. The nation consistently ranks among those countries with the highest amounts of antibiotics consumed per 1000 residents and exhibits high levels of resistance to drugs that are considered critical in treating patients such as carbapenems and fluoroquinolones [34]. Resistant pathogens which are present in intensive care units (ICUs) and operating rooms frequently result in hospital-acquired infections where viable treatment alternatives are limited [35]. While initiatives such as the National Action Plan GR-AMR and the strengthened role of the Hellenic National Public Health Organization (EODY) have been introduced, surveillance and policy enforcement remain substantially insufficient.

India is indicative of a developing country experiencing a significant amount of burden in terms of its epidemiologic prevalence due to AMR [36]. Overuse and overprescribing of antibiotics and the lack of proper regulation regarding prescriptions for antibiotics have resulted in the emergence of highly resistant strains, such as NDM-1 (New Delhi metallo-beta-lactamase) [37]. In 2020, more than half of the isolated *Escherichia coli* strains in the country showed resistance to at least three different antibiotic classes [38]. In addition to unregulated prescription practices, India also has issues with counterfeit medicine circulation and poor oversight of antibiotic usage in agriculture.

Beyond the United States, Europe, Greece and India, the global AMR burden is substantial in Africa, Latin America, Southeast Asia and the Middle East. In the WHO African Region, mortality rates attributable to bacterial AMR are among the highest globally, but many countries face limited laboratory capacity and incomplete surveillance coverage [30,39]. In Latin America and the Caribbean, deaths associated with resistant infections are driven by resistant *Enterobacterales*, *Acinetobacter baumannii* and *Staphylococcus aureus*, with large differences in diagnostic and stewardship capacity across countries [40,41]. South and Southeast Asia continue to report high resistance among *Escherichia coli*, *Klebsiella pneumoniae* and other Gram-negative pathogens, often in the context of broad antimicrobial access, variable prescription control and environmental contamination [30,36,37,38]. In the Eastern Mediterranean and Middle East, WHO-GLASS data indicate high resistance signals for several priority pathogen–antibiotic combinations, while conflict, displacement and fragmented health systems can further weaken infection prevention and surveillance [30].

A comparative global snapshot illustrates that AMR presents challenges that are both complex and multifaceted. Areas with robust surveillance systems, educated populations, effective infection-prevention programs and established stewardship policies tend to detect and respond to AMR more effectively [42], whereas countries with ineffective regulatory frameworks, limited access to quality care, environmental contamination and uncontrolled antimicrobial use appear to be at the epicenter of the current crisis [43] (Table 1).

Several useful tools have been developed to help analyze trends and assess epidemiologic risk associated with AMR, such as WHO’s GLASS platform, One Health Trust’s ResistanceMap and ECDC’s interactive maps [45,46,47]. However, the reliability of such data depends on a strong commitment from national governments to develop functional surveillance systems capable of providing accurate information. At present, many countries lack well-developed surveillance systems that hinder the global understanding of AMR.

Overall, AMR epidemiological data play a crucial role in informing the development of prevention strategies. Nevertheless, their effectiveness depends on continuous updating, improved transparency, and sustained international collaboration.

## 5. Consequences of Antimicrobial Resistance

A major concern about AMR is that its effects extend beyond the narrow framework of clinical medicine. Public health, global economy and social cohesion are all negatively affected. The progressive loss of antibiotic effectiveness leads to higher morbidity and mortality, burdens on health systems, and deeper social inequalities [41,48,49].

At the public health level, AMR dramatically reduces therapeutic effectiveness against common and previously controllable infections, such as pneumonia and urinary tract infections. Treatments become more complex, more toxic and less successful, resulting in more complications and longer hospital stays. This leads not only to a deterioration in the patients’ quality of life but also to an increased risk of death, especially among vulnerable groups such as older adults, immunocompromised individuals and patients in ICUs [50,51,52]. AMR also undermines the safety of critical medical procedures that depend on effective infection prevention and treatment, such as surgeries, transplants, chemotherapy and the care of premature newborns [53,54].

The economic consequences of AMR are equally significant and are expected to intensify in the coming years. The need for longer hospital stays, more expensive treatments and more specialized diagnostic tests dramatically increases healthcare costs. At a global level, it is estimated that, without decisive intervention, AMR could lead to a reduction in global Gross Domestic Product (GDP) of 2% to 3.5% by 2050, with total economic damage exceeding 100 trillion USD [55,56].

At the social level, AMR exacerbates existing inequalities and disproportionately affects poorer and more marginalized communities. In many low- and middle-income countries, populations are more frequently exposed to resistant pathogens because of inadequate infrastructure, lack of clean water and sewerage systems and limited access to basic health services and medicines [44,57].

Overall, the consequences of AMR create a vicious cycle in which worsening public health, economic strain and social insecurity reinforce one another, making the problem increasingly severe and harder to reverse. Its effective management requires not only technological progress but also institutional, economic and social mobilization.

## 6. Bioethical Dimensions of Antimicrobial Resistance

The AMR crisis is a field where ethical, political and social dilemmas collide. Bioethics helps clarify competing duties concerning the right to health, equity in access to treatment, professional responsibility, state obligations and protection of future generations. Established frameworks are useful for structuring this analysis. Principlism highlights beneficence, non-maleficence, and respect for autonomy and justice; utilitarian public-health ethics emphasizes maximizing population benefit and preventing avoidable harm; and global justice frameworks draw attention to the unfair distribution of AMR risks, resources and decision-making power between high-income and low- and middle-income countries [58,59,60,61].

The core of this issue is justice. AMR exacerbates existing inequalities between the rich and the poor on a national level, as well as among different social groups on a local level [49]. Citizens living in high-income countries generally have greater access to safe medicines, health services and surveillance programs, while in low- and middle-income countries, self-medication, the use of counterfeit or expired drugs and the absence of basic services lead to the uncontrolled spread of resistant pathogens [62,63]. This creates an ethically unacceptable situation in which place of residence and socioeconomic background may determine the outcome of an infection. Furthermore, residents of low- and middle-income countries, although they bear the greatest burden of AMR, are generally excluded from decision-making processes regarding international health policy, which deepens the deficit in representation and moral responsibility. This justice problem is visible, for example, when patients in low-resource settings face simultaneous risks of restricted access to effective antibiotics, limited microbiological diagnostics, substandard or counterfeit medicines and higher exposure to resistant pathogens through inadequate water and sanitation infrastructure [23,24,44,57,62,63].

Another significant bioethical aspect of this problem is the balance between individual freedom and collective responsibility [58]. The use of antibiotics is an action that affects individuals but also has consequences for others. When a patient takes an antibiotic without medical indication or fails to complete the course of treatment, they not only risk their own health but also contribute to the emergence and spread of drug-resistant bacteria affecting the entire population [64]. Similarly, physicians who prescribe antibiotics under pressure from patients fail to consider the negative impact on future generations. Consequently, AMR highlights how far individual actions can or should be regulated when the collective good of health is at stake. From a principlist perspective, stewardship can be understood as an attempt to balance individual benefit and autonomy with non-maleficence toward other patients and future patients who may be harmed by resistance generated today [58,59,60,64].

Antimicrobial stewardship policies therefore raise specific ethical questions. Restrictions on broad-spectrum or last-resort antibiotics may be justified when they are evidence-based, transparent, proportionate and accompanied by rapid diagnostics, expert consultation and timely access for patients who genuinely need treatment. However, stewardship becomes ethically problematic if it is implemented as simple rationing in settings where patients already lack access to healthcare, quality-assured medicines, microbiology laboratories, or affordable follow-up. Ethical stewardship must therefore pursue two goals simultaneously: reducing unnecessary antimicrobial exposure and ensuring equitable access to effective therapy for those in need, particularly in low- and middle-income countries [23,24,59,62,63].

A third important ethical issue is intergenerational justice [59,60]. The irrational use of antimicrobials today poses a serious threat to the ability of future generations to prevent or treat even the most common infections. Thus, future generations who were not involved in the current generation’s decisions may be denied one of the most basic tools of modern medicine. Therefore, AMR represents not just a present crisis but also an ethical injustice toward the future, much like environmental destruction and, thus, there is a moral responsibility to preserve effective antimicrobials for future generations [59,60].

In addition, the stance of the pharmaceutical industry and the responsibilities placed upon nation-states raise important ethical concerns [58,59]. Pharmaceutical companies have greatly diminished research and development efforts for new antibiotics, largely due to lower profitability compared to chronic medications or “everyday-use” medications. This prioritization of profit over public health underscores the ethical concern surrounding corporate responsibility and the regulation of pharmaceutical innovation. Likewise, governments have moral duties for failing to develop appropriate policies to address misuse of antibiotics, as well as for promoting international cooperation necessary to confront this transnational problem.

In summary, AMR is an area where medicine, ethics, public health, economics and politics converge in complex ways. Understanding AMR requires not only scientific knowledge but also ethical judgment so that decisions today account for clinical benefit, public health protection, social justice, transparency and intergenerational solidarity.

## 7. Alternative Therapeutic Approaches to Infections Without the Use of Antibiotics

The rapid rise of AMR has made it imperative to explore therapeutic and preventive approaches beyond conventional antibiotics. Recognizing the gravity of the situation, the global scientific community has focused on bacteriophages, antimicrobial peptides, microbiome-based strategies, immunotherapy, vaccination and nanotechnology [65,66,67,68,69,70]. These approaches should not be presented only as solutions; their clinical value depends on evidence quality, regulatory feasibility, safety, cost, scalability and integration with stewardship and diagnostics (Table 2).

Bacteriophage therapy is one of the most discussed alternatives to traditional antibiotics. Bacteriophages selectively target bacteria and can be adapted in the laboratory to match a patient-specific pathogen [71]. This targeted action may limit off-target effects and preserve the normal microbiota. However, the same specificity also creates implementation challenges: clinicians need rapid pathogen identification, phage-susceptibility testing, access to characterized phage libraries and standardized manufacturing. Regulatory pathways remain difficult because personalized phage mixtures do not fit neatly into conventional approval models for fixed pharmaceutical products and more controlled clinical data are needed to define efficacy, dosing, pharmacokinetics, immune interactions and resistance risk [72,73].

Antimicrobial peptides are another promising field. These peptides, found in plants, animals and humans, can disrupt microbial membranes and may also modulate innate immune responses [74,75]. Nevertheless, translation into routine therapy remains difficult. Many peptides are rapidly degraded by proteases, have short half-lives and may require specialized delivery systems. Toxicity, hemolysis, immunogenicity and high production costs further limit clinical use, meaning that many candidates remain in preclinical or early clinical development rather than established treatment options [75,76].

Microbiome-based interventions and probiotics aim to restore colonization resistance after dysbiosis caused by illness or antibiotic exposure [77,78,79,80]. Their role is best understood as preventive or supportive rather than as a full substitute for antimicrobial therapy. Evidence is strongest for selected settings, such as prevention of antibiotic-associated diarrhea, but clinical efficacy is inconsistent across strains, doses, patient groups and infection outcomes. Safety and quality-control issues also matter, particularly for immunocompromised patients or products that differ substantially in composition and manufacturing standards.

Immunotherapeutic strategies include vaccines, monoclonal antibodies and other tools that strengthen host defense [81,82,83]. Vaccines are especially important as preventive tools against AMR because they reduce infection incidence, decrease antibiotic prescribing and can interrupt transmission of resistant pathogens [82,84]. Some vaccines are already clinically established for bacterial diseases, whereas other AMR-relevant candidates remain in clinical or preclinical development. Monoclonal antibodies may provide targeted adjunctive therapy, but cost, pathogen specificity, access and the need for clear clinical endpoints can limit widespread implementation [81,83].

Nanotechnology offers opportunities for targeted antimicrobial delivery, antibiofilm strategies and materials with direct antimicrobial activity [85,86,87]. Metallic nanoparticles can generate reactive oxygen species and disrupt bacterial membranes, while nanoparticle carriers may deliver existing drugs more precisely to infection sites [85,86,87]. However, long-term safety, biodistribution, environmental persistence, reproducibility, scale-up, standardization, cost and regulatory approval remain unresolved barriers. For this reason, nanotechnology should be viewed as a promising but still largely translational platform rather than an immediately deployable replacement for antibiotics.

The development of alternative approaches is a critical component of the long-term global response to AMR, but these strategies are most realistic when integrated with antibiotics, diagnostics, infection prevention and stewardship rather than framed as complete replacements. Their success will depend on rigorous clinical trials, harmonized regulation, sustainable financing, equitable access and public and professional acceptance.

## 8. Conclusions

AMR is one of the most serious multifactorial problems of our time, with continuously intensifying consequences for global health, society and the economy. The development of new antibiotics cannot keep pace with the rapid emergence of antibiotic-resistant microorganisms; thus, AMR is systematically undermining the achievements of modern medicine and potentially threatening to return humanity to a pre-antibiotic era in which even simple infections could prove life-threatening.

The examination of the causative factors reveals that AMR is not simply a biological phenomenon. Rather, it is the result of collective behaviors, institutional omissions and inequalities on a global scale. The widespread misuse of antibiotics, inadequate surveillance, lack of political will to address AMR, unequal access to quality care and insufficient international cooperation contribute to creating environments conducive to continued spread of resistant microorganisms. There are considerable variations within and among countries concerning their epidemiological and political responses to AMR. Therefore, it is essential to implement regionally specific, tailored interventions while establishing a unified, global framework.

Addressing AMR necessitates a paradigm shift in thinking that combines public health with bioethics, scientific advancement with justice and international cooperation with local implementation. Redefining our relationship with antibiotics through public education, market regulation, and stronger healthcare systems are strategies of decisive importance. Surveillance and “rational antibiotic use” programs must be integrated as institutional tools into national health systems, not as emergency measures but as permanent policies. In conclusion, AMR is one of the challenges that requires a collective response. Overcoming it is not only a matter of technical solutions, but a deeply ethical and political imperative. Protecting the effectiveness of antimicrobial drugs is the responsibility of all and an inalienable right of both present and future generations.

## 9. Future Prospects

AMR is dynamic and evolves under the influence of global changes such as the climate crisis, urbanization, international population movements, conflict and shifts in food systems. Future projections indicate that resistant bacterial infections will remain a major cause of mortality and economic loss unless prevention, treatment access and innovation improve substantially [8]. The future response should therefore combine classical public health measures with the Environmental, Social and Governance (ESG) pillars: environmental stewardship to reduce antimicrobial dissemination, social equity to ensure fair access to diagnostics and treatment, and accountable governance to strengthen stewardship, surveillance and policy implementation.

Artificial intelligence (AΙ)-assisted antimicrobial discovery is likely to become increasingly important for identifying new molecular frameworks for antimicrobial development, repurposing existing compounds, predicting resistance mechanisms and prioritizing molecules for laboratory validation [88,89]. However, AI-generated candidates require careful experimental confirmation, toxicity assessment and stewardship planning to avoid repeating the cycle of rapid resistance emergence. Rapid molecular diagnostics and next-generation antimicrobial susceptibility testing can also improve patient care by shortening the time from specimen collection to targeted therapy, thereby reducing unnecessary broad-spectrum antibiotic exposure [90].

Genomic surveillance, metagenomics and integrated data systems are central to future AMR control. Whole-genome sequencing can identify transmission clusters, resistance determinants and mobile genetic elements, while metagenomics may detect resistance genes in wastewater, hospitals, animals and community environments before clinical outbreaks become apparent [28,91]. Precision-medicine approaches should link local antibiograms, patient risk factors, pathogen genomics, rapid diagnostics and stewardship decision support to select the narrowest effective treatment at the right dose and duration. The key challenge will be ensuring that these technologies are affordable, interoperable, ethically governed and available beyond high-income settings.

## Figures and Tables

**Table 1:** Comparative global snapshot of antimicrobial resistance.

Setting/WHO Region	Burden and Mortality Indicator	Major Resistance Pathogens and Trends	Antibiotic-Use and Surveillance Context
United States/Region of the Americas	>2.8 million resistant infections annually; at least 35,000 deaths [31].	*Candida auris* and carbapenem-resistant *Enterobacterales*. MRSA and other urgent or serious threats remain important [31].	Extensive CDC surveillance and stewardship infrastructure; continuing need for antibiotic-consumption monitoring and infection prevention [32].
European Union/European Region	EU burden remains uneven; Greece has very high antibiotic consumption and high resistance to carbapenems and fluoroquinolones [33,34,35].	*Klebsiella pneumoniae* and *Acinetobacter baumannii* are major hospital threats, especially in ICUs and surgical wards [33,34,35].	EARS-Net and national action plans support monitoring; implementation and enforcement gaps persist in several settings [33,34,35].
Sub-Saharan Africa/African Region	Among the highest AMR-attributable mortality rates globally in 2019, with under-detection due to limited laboratory capacity [39].	Resistant Gram-negative bacteria, invasive non-typhoidal *Salmonella*, *Streptococcus pneumoniae* and *Staphylococcus aureus* are important contributors [39].	Surveillance coverage, diagnostics, WASH and access to quality-assured antibiotics remain major constraints [30,39,44].
Latin America and the Caribbean/Region of the Americas	Substantial AMR-associated and attributable mortality was estimated in 2019, with large country-to-country variation [40,41].	Resistant *Enterobacterales*, *Klebsiella pneumoniae*, *Acinetobacter baumannii* and MRSA are recurrent concerns [40,41].	Stewardship and laboratory capacity are heterogeneous; over-the-counter access and unequal healthcare access remain challenges in some settings.
South and Southeast Asia/South-East Asia and Western Pacific regions	High burden, especially in populous settings, with frequent resistance in *Escherichia coli* and *Klebsiella pneumoniae* [30,36,37,38].	ESBL-producing *Enterobacterales*, NDM-producing carbapenem-resistant *Enterobacterales* and other multidrug-resistant Gram-negative pathogens are key threats [36,37,38].	High antimicrobial consumption, variable prescription control, counterfeit/substandard medicines and environmental contamination require One Health action [30,36,37,38].
Middle East and North Africa/Eastern Mediterranean Region	WHO-GLASS highlights high resistance signals in several pathogen–antibiotic combinations, although data completeness varies [30].	Carbapenem-resistant *Enterobacterales, Acinetobacter baumannii* and ESBL-producing organisms are major hospital concerns [30].	Conflict, displacement, fragmented health systems and variable laboratory infrastructure can weaken surveillance and infection prevention [30].

EARS-Net: European Antimicrobial Resistance Surveillance Network; ESBL: Extended-Spectrum Beta-Lactamase; NDM: New Delhi metallo-beta-lactamase; MRSA: Methicillin-resistant *Staphylococcus aureus*; WASH: Water, Sanitation and Hygiene; WHO-GLASS: WHO–Global Antimicrobial Resistance and Use Surveillance System.

**Table 2:** Major non-antibiotic therapeutic and preventive approaches.

Approach	Main Mechanism and Current Stage	Potential Strengths	Limitations, Regulatory Status and Evidence
Bacteriophage therapy	Viruses selectively infect and lyse specific bacteria [71]. Used mainly in compassionate, personalized, or specialized settings; clinical trials are ongoing [72,73].	Highly targeted action; may preserve normal microflora; can be adapted to pathogen susceptibility [72,73].	Regulatory pathways remain challenging because individualized mixtures, rapid pathogen identification, susceptibility testing, GMP manufacturing, pharmacokinetics, immune responses and phage resistance must be addressed. Evidence is promising but still heterogeneous.
Antimicrobial peptides	Natural or synthetic molecules disrupt microbial cell membranes or modulate host immunity [74]. Many candidates remain preclinical or early translational [75,76].	Broad activity against some multidrug-resistant microbes; potential synergy with existing antimicrobials [75].	Protease degradation, short half-life, delivery difficulties, manufacturing costs and toxicity concerns limit clinical translation [76].
Probiotics and microbiome-based strategies	Restore protective microflora and limit pathogen colonization through ecological inhibition [77,78,79,80]. Used as supportive/preventive tools in selected indications.	May reduce antibiotic-associated dysbiosis and support prevention in defined contexts, especially after disruption of normal flora.	Clinical efficacy is strain-, dose-, host- and indication-specific. Results are inconsistent across trials; product quality control and safety in immunocompromised hosts require caution.
Immunotherapy and antimicrobial vaccines	Vaccines, monoclonal antibodies and targeted immune tools prevent infection or strengthen host response [81,82,83,84].	Vaccines can reduce infection incidence and antibiotic demand; antibodies may offer pathogen-specific adjunctive treatment.	Pathogen specificity, antigenic diversity, cost, access and trial endpoints complicate development. Some vaccines are established, but many vaccines targeting antibiotic-resistant microbes remain in development.
Nanotechnology-based therapies	Nanoparticles may act directly against microbes or improve targeted drug delivery [85,86,87]. Mostly preclinical or early translational, with limited approved antibacterial indications.	May enhance local efficacy, reduce systemic dosing and enable delivery of combined antimicrobial strategies.	Long-term safety, biodistribution, environmental effects, reproducibility, scale-up, standardization, cost and regulatory approval remain major barriers [87].

GMP: Good Manufacturing Practices.

## Data Availability

No new data were created or analyzed in this study. Data sharing is not applicable to this article.

## Abbreviations

The following abbreviations are used in this manuscript:

AMR	Antimicrobial resistance
CDC	Centers for Disease Control and Prevention
ECDC	European Centre for Disease Prevention and Control
EODY	Hellenic National Public Health Organization
EU	European Union
GDP	Gross Domestic Product
GLASS	Global Antimicrobial Resistance and Use Surveillance System
ICUs	Intensive care units
NDM-1	New Delhi metallo-beta-lactamase
WASH	Water, sanitation and hygiene
WHO	World Health Organization
